# Evaluation of the fetal abdomen by magnetic resonance imaging. Part 2: abdominal wall defects and tumors

**DOI:** 10.1590/0100-3984.2016.0142

**Published:** 2018

**Authors:** Ana Paula Pinho Matos, Luciana de Barros Duarte, Pedro Teixeira Castro, Pedro Daltro, Heron Werner Júnior, Edward Araujo Júnior

**Affiliations:** 1 MD, Physician in the Radiology Department of the Clínica de Diagnóstico Por Imagem (CDPI), Rio de Janeiro, RJ, Brazil.; 2 PhD, Adjunct Professor in the Department of Maternal-Infant Care of the Universidade Federal Fluminense (UFF), Niterói, RJ, Brazil.; 3 MSc, MD, Physician in the Radiology Department of the Clínica de Diagnóstico Por Imagem (CDPI), Rio de Janeiro, RJ, Brazil.; 4 PhD, MD, Physician in the Radiology Department of the Clínica de Diagnóstico Por Imagem (CDPI), Rio de Janeiro, RJ, Brazil.; 5 Tenured Adjunct Professor in the Department of Obstetrics of the Escola Paulista de Medicina da Universidade Federal de São Paulo (EPM-Unifesp), São Paulo, SP, Brazil.

**Keywords:** Fetus, Congenital abnormalities/diagnostic imaging, Abdomen/diagnostic imaging, Magnetic resonance imaging

## Abstract

Although ultrasound is still the gold standard for the assessment of fetal malformations, magnetic resonance imaging (MRI) has gained great prominence in recent years. In situations in which ultrasound has low sensitivity, such as maternal obesity, abdominal scarring, and oligohydramnios, MRI has proven to be a safe and accurate method. Regarding fetal abdominal wall defects, MRI appears to be widely used in the prognostic assessment of gastroschisis with intestinal atresia or of complications of omphalocele, allowing better perinatal management and parental counseling. In addition, MRI allows the assessment of local invasion of fetal abdominal tumors, with significant prognostic value for the postnatal period. In this article, we review the main MRI findings in the evaluation of fetal abdominal wall defects and tumors.

## INTRODUCTION

The importance of imaging methods in the diagnosis of congenital malformations^(^^1^^-^^3^^)^, and especially in fetal medicine^(^^4^^-^^7^^)^, has been the objective of a series of recent studies conducted in Brazil. Ultrasound continues to be the preferred method for evaluating fetal malformations because of its wide acceptance, low cost, and low risk for the mother and fetus. However, in certain conditions, such as maternal obesity, excessive fetal movement, abdominal scarring, and diminished amniotic fluid volume, ultrasound has low sensitivity.

Magnetic resonance imaging (MRI) has been applied in situations in which ultrasound has low sensitivity, showing advantages in the evaluation of some malformations, such as cortical maturation disorders^(^^8^^)^. With respect to malformations in the fetal abdominal wall, MRI is typically used in the prognostic evaluation of intestinal atresia, gastroschisis, and omphalocele complications, permitting better perinatal management and parental counseling^(^^9^^)^. MRI is a more accurate method than is ultrasound for the characterization of the pelvic and abdominal extent of sacrococcygeal tumors^(^^10^^)^, as well as providing more information on the compression of adjacent organs.

In this paper, we present MRI findings of the principal malformations of the fetal abdominal wall in the form of a pictorial essay.

## ABDOMINAL WALL DEFECTS AND TUMORS

### Omphalocele

Omphalocele is a defect of the anterior abdominal wall with encapsulation by the parietal peritoneum and herniation of the abdominal contents, resulting from failed migration of the lateral body^(^^11^^)^. Omphalocele occurs in 1/4000 live births^(^^12^^)^ and is accompanied by other malformations in 72% of those births. It is also related to chromosome disorders, trisomies 18 and 13, which worsen the prognosis, being present in 30-40% of cases. The disorders most often accompanying omphalocele are those of a cardiac, genitourinary, gastrointestinal, or musculosketal nature, as well as defects of the neural tube, head, or neck^(^^13^^)^.

**Figure 1:**
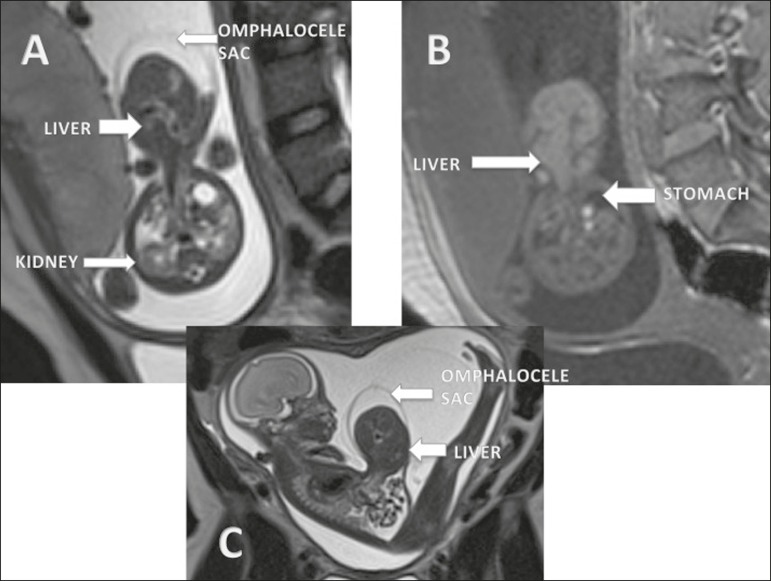
Omphalocele in a fetus at 22 weeks. **A:** Axial T2-weighted sequence showing a hernia sac with herniation of the liver. **B:** Axial T1-weighted sequence showing the hepatic herniation. **C:** Sagittal T2-weighted sequence showing an abdominal wall defect with a hernia sac containing the liver.

In prenatal screenings, omphalocele is successfully diagnosed with ultrasound in 66-93% of cases. An MRI examination adds additional anatomic details in the evaluation of omphalocele. It shows the hernia sac, the central defect of the abdominal wall with herniated abdominal viscera surrounded by a thin capsule formed by the Wharton's jelly, which separates the abdominal contents from the amniotic fluid. The volume and its contents (liver, stomach, spleen, and colon) are variable. MRI demonstrates the central abdominal defect and herniated viscera, a thin capsule surrounding its contents and separating them from the amniotic fluid (Figure 1). The liver can be seen as a large, solid hypointense mass in T2-weighted sequences.

When omphalocele is accompanied by other malformations, the mortality rate is 80% and reaches 100% in individuals with a chromosome disorder. In cases of omphalocele without other malformations or chromosome disorders, perinatal mortality is only 19%, revealing the importance of careful fetal morphologic evaluation for maternal counseling. The presence or absence of the liver in the hernia sac does not alter the prognosis^(^^14^^)^.

### Gastroschisis

Gastroschisis is the herniation of the abdominal contents through a defect in the periumbilical abdominal wall on the right. It is a more rare anomaly than is omphalocele, with an incidence of 1/10,000 to 6/10,000 live births^(^^15^^)^. Unlike omphalocele, there is no hernia sac in gastroschisis and the abdominal contents are in direct contact with the amniotic fluid. Gastroschisis is accompanied by other malformations in 19-31% of cases, the most common being intestinal malformations such as atresia and stenosis. It presents complications similar to those of other intestinal anomalies, such as intestinal obstruction, perforation, peritonitis, necrotizing enterocolitis, short bowel syndrome, and fistulas.

Although the prevalence of gastroschisis has increased in recent years, the development of parenteral nutrition techniques has reduced mortality rates from 60% in the 1960s to 3-10% in recent years, the principal prognostic factor being intestinal integrity^(^^16^^)^.

**Figure 2:**
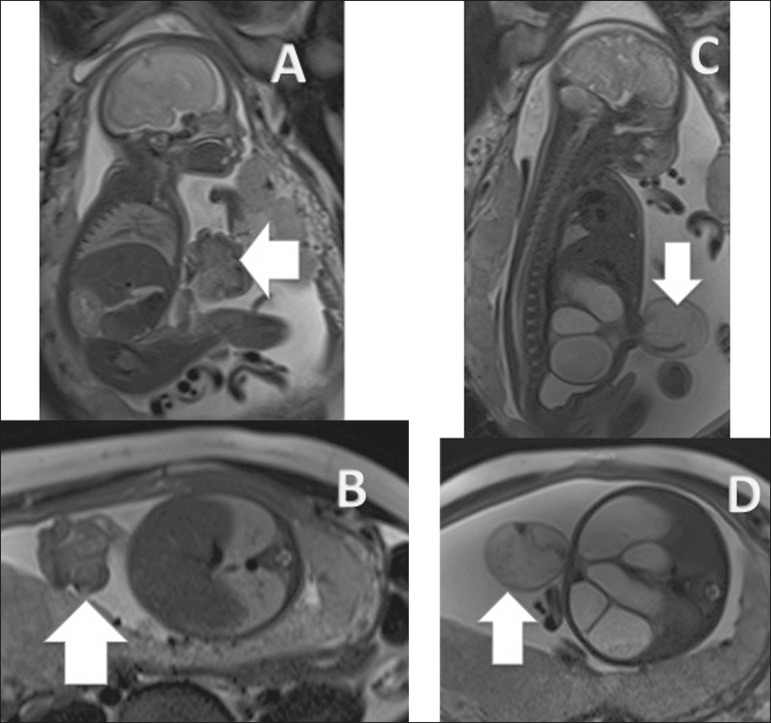
Gastroschisis. **A,B:** Sagittal T2-weighted sequence of a fetus at 27 weeks, showing an abdominal wall closure defect and intestinal herniation (arrows). **C,D:** Sagittal and axial T2-weighted sequences of the fetus at 33 weeks showing gastroschisis. Intestinal herniation with pronounced dilation of the intestinal loops, simulating a hernia sac (arrows).

The correct evaluation of intestinal integrity is fundamental to the neonatal prognosis of gastroschisis. An MRI study shows intestinal loops herniated through the intestinal wall, floating freely in the amniotic fluid. The abdominal wall defect presents to the right of the umbilical cord insertion. Dilation and thickening of the loops could indicate abnormalities such as atresia and necrosis (Figure 2). The differential diagnoses are omphalocele rupture, herniation of the umbilical cord, and body stalk anomaly. The presence of solid structures floating freely indicates omphalocele rupture. The prognosis of gastroschisis is related to the bowel conditions at birth and to the presence of other malformations. Dilation larger than 17 mm and thickening of the loops of more than 3 mm can be related to high morbidity, as can polyhydramnios.

### Pentalogy of Cantrell

First described in 1958, pentalogy of Cantrell is a combination of malformations in the abdominal wall, sternum, diaphragm, pericardium, and heart. It is a rare malformation, only a few hundred cases having been reported, and is more common in male fetuses. The malformations characteristic of the syndrome are midline, supraumbilical defects in the abdominal wall; defects in the caudal part of the sternum; malformation of the anterior diaphragm; and congenital heart disease.

**Figure 3:**
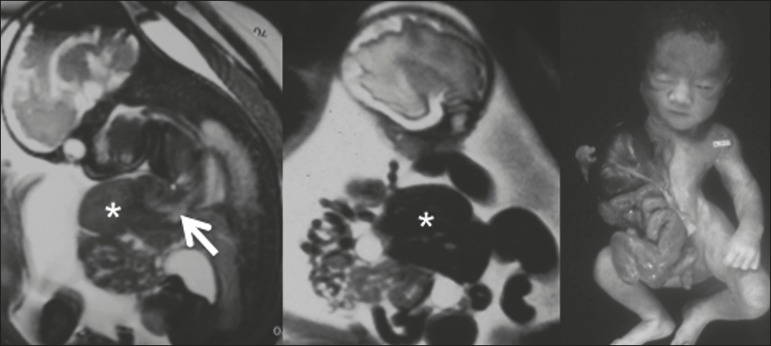
Pentalogy of Cantrell in a fetus at 27 weeks. Note protrusion of the liver (asterisk), heart (arrow), and intestinal loop through anterior defects in the thoracic and abdominal walls.

In fetuses with pentalogy of Cantrell, the most common abdominal wall defect is omphalocele. A cleft in the lower part of the sternum, with herniation of the heart, characterizes malformation of the sternum. In 91% of cases, the defect is in the anterior diaphragm. Contiguity between the pericardial and peritoneal cavities is common. Among cardiac defects, significant and complex congenital heart diseases are the rule. The malformations most often accompanying pentalogy of Cantrell are those involving the thoracic and abdominal organs. Craniofacial and lower limb defects are also common (seen in 28% of cases). MRI shows omphalocele, ectopic heart, pleural effusion, and pericardial effusion. As shown in Figure 3, defects of the diaphragm can be difficult to assess by MRI^(^^17^^)^.

### Body stalk anomaly

**Figure 4:**
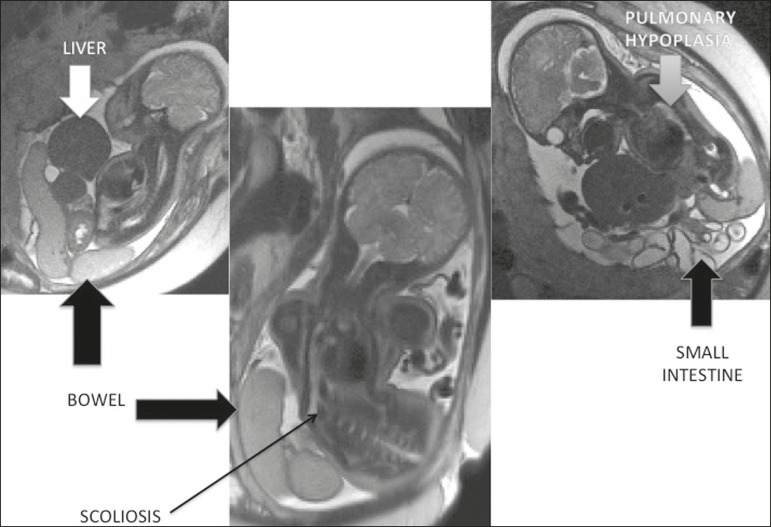
Body stalk anomaly. T2-weighted sequences showing herniation of the liver and intestine, with pulmonary hypoplasia and scoliosis.

Body stalk anomaly consists of a variable group of malformations of the lower limbs and pelvic girdle, accompanied by defects in the abdomen and thorax^(^^12^^)^. With an incidence of 1/14,000 live births, its etiopathogenic factors include amniotic band syndrome and abrupt interruption of vascularization during embryonic development. The malformations most often described are abdominoschisis, which are typically left-sided and voluminous; defects in the thoracic wall; abnormal rotation of the lower limbs; congenital talipes equinovarus (clubfoot); brachydactyly; polydactyly; syndactyly; absence of lower limbs; and scoliosis (Figure 4). Body stalk anomaly is associated with facial and cranial defects such as exenphaly, encephalocele, and facial defects. Myelomeningocele, with hydrocephalus and Arnold-Chiari malformation, can also be present. Among fetuses with body stalk anomaly, there is a high incidence of cardiac and diaphragmatic malformations. The umbilical cord is short or absent, the fetal thorax and abdomen adhering to the placenta, and there is herniated viscera. Amniotic band syndrome is seen in 40% of cases.

In cases of body stalk anomaly, MRI shows the placenta without evidence of an umbilical cord. Abdominal, thoracic, lower limb, craniofacial, and internal organ anomalies have a variety of presentations. At times, the herniated organs form a complex mass The amniotic band can be identified as a linear structure.

## SACROCOCCYGEAL TERATOMA

Sacrococcygeal teratomas are tumors of embryonic origin resulting from the development and proliferation of pluripotent cells. The diagnosis is usually made in routine prenatal examinations. Neonatal mortality approximates 50% in cases of hydrops fetalis and prematurity.

**Figure 5:**
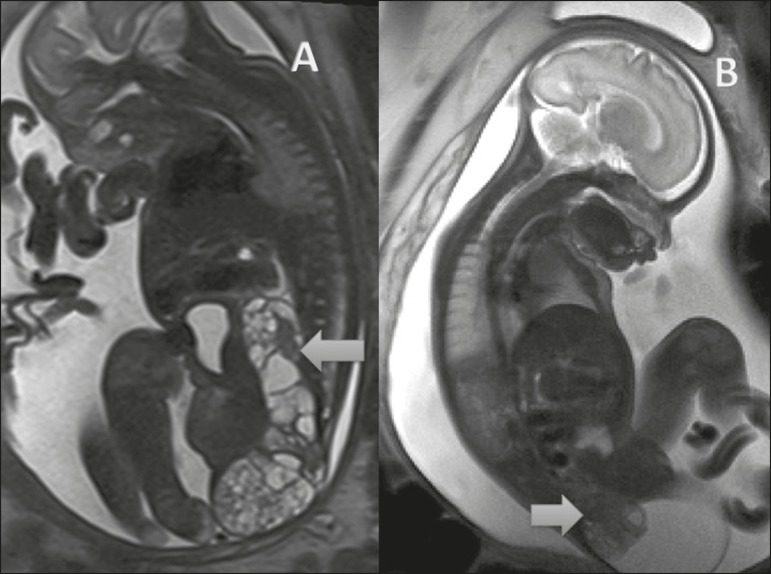
Sacrococcygeal teratoma. **A:** Sagittal T2-weighted sequence showing a type III sacrococcygeal teratoma extending to the pelvis and abdomen in a fetus at 24 weeks (arrow). **B:** Sagittal T2-weighted sequence showing a type II sacrococcygeal teratoma in a fetus at 30 weeks (arrow).

MRI helps assess the extent of a sacrococcygeal teratoma, the involvement of adjacent organs, and the compressive effect of the lesion (Figure 5). During the prenatal period, MRI also evaluates the volumetric growth of the teratoma, which, together with the Doppler velocimetry study, classifies the disease as high or low risk. Because of the high morbidity and mortality associated with sacrococcygeal teratoma, surgery during gestation is a promising treatment^(^^18^^)^.
